# Supplementary material for: Are carbon emissions trading and green financial instruments synergistic? -Comprehensive quantitative research based on content analysis
Source: PLoS One. 2024 Mar 7;19(3):e0298601. doi: 10.1371/journal.pone.0298601 (PMC10919601; doi:10.1371/journal.pone.0298601)
Supplement: S1 Data — (ZIP) [file pone.0298601.s001.zip › Data/地级市Z_F工作报告.zip!/╝¬┴╓/░╫│╟╩╨/└┤╫╘╣1⁄2╓┌║┼í╛┬φ┐╦╩2╛▌═°í┐_2016.docx]

各位代表：

　　现在，我代表市政府，向大会报告政府工作，请予审议，并请市政协各位委员提出意见。

　　一、2015年工作回顾

　　一年来，我们在省委、省政府和市委的正确领导下，在市人大、市政协的监督支持下，紧紧依靠全市人民，主动适应发展新常态，积极应对经济下行压力，加快建设吉林西部生态经济区，推动白城经济社会发展取得了新成效，为“十二五”收官、“十三五”开局奠定了坚实基础。预计全市地区生产总值完成740亿元，增长7.5%。固定资产投资完成670亿元，增长12%。规模以上工业增加值完成210亿元，增长8%。地方级财政收入完成39.5亿元，下降4.6%。社会消费品零售总额完成318亿元，增长12%。城镇和农村常住居民人均可支配收入分别为19600元、7890元，均增长8%，与经济保持同步增长。

　　——经济运行保持平稳。项目招商成效明显，实施3000万元以上项目366个，亿元以上项目250个，“百日攻坚”活动成果显著。“民企吉林行”、“央企走进吉林”和“东博会”签约项目191个。全市招商引资到位资金983亿元、增长20%。工业运行态势良好，新增规模以上企业48户。“五业振兴”加快推进，五大重点产业增加值占比达到80%以上。开发区转型升级步伐加快。“富民强村固基”工程深入实施，农业生产稳中有升，粮食产量再创历史新高。“六畜兴旺”全面推进，畜禽饲养总量达到5250万头（只）、增长11.4%。洮南被确定为国家现代农业示范区、循环经济示范市。服务业加快发展，上海西郊国际农产品展销中心“白城馆”开馆运营。通榆列入全国电子商务进农村综合示范试点县。全市电子商务交易额3.7亿元。金塔集团、风雷公司成功在“新三板”挂牌。“一线八点”精品旅游线路加快建设，旅游业收入达到46亿元，增长20%。外贸出口转型示范基地加快建设。服务业增加值完成270.3亿元，增长7.4%。民营经济快速壮大，民营企业达到8450户，增长25.6%；主营业务收入1157亿元，增长7.9%。

　　——重点改革深入推进。简政放权力度加大，成立职能转变协调办公室，政府承担的45项改革任务有效推进。40个部门建立权力清单。政府部门行政职权减少1492项，行政审批项目减少37项，非行政许可审批全部取消。“五证合一”全面推行，工商登记前置改后置审批151项。完成公务员工资改革和津贴补贴调整。县级公立医院综合改革全面铺开。6户“粮食银行”挂牌运行。农村金融综合改革试验区被列为全国唯一市级试点，组建市级物权融资公司、农村产权交易中心，大安农商行、洮南惠民村镇银行、洮北惠民村镇银行、通榆农商村镇银行正式挂牌运营。通榆、镇赉列入国家“粮改饲”试点。安广镇纳入全省重点城镇扩权试点。探索组建大学区模式，校长教师交流轮岗试点有序开展。电力市场交易、“农易保”融资等试点有效开展。我市被确定为全国社会保障卡综合应用试点示范市。“通榆县服务城乡群众一张网”改革做法入选中央改革办《改革案例选编》。

　　——城市建设步伐加快。我市成功列入全国“海绵城市”试点，争取国家专项补助资金12亿元，开工项目101项。启动地下综合管廊建设。开展城市建设管理三年攻坚战。老城区实施工程8项，改造街路17.7公里，新建改造污雨水管网52公里、二次供水管线31.6公里、燃气管网20公里。生态新区道路水系、绿化景观、新城家园等工程基本完工，妇幼保健院、洮北疾控中心竣工，市民服务中心、九年制学校项目加快推进。林海镇?生态新区纳入第二批国家新型城镇化试点。生态新区被列为全省低碳社区试点。城际互联互通基础设施建设取得重大进展，嫩丹高速、白阿铁路、长白铁路加快推进，长庆、丽江、长白立交桥开工建设。白城机场整体竣工，机场连接线实现通车。镇赉县荣获“中国宜居城镇”称号。

　　——生态环境明显改善。启动水生态文明城市试点项目35个。河湖连通、月亮泡蓄滞洪区、嫩江干流治理等6个重点项目，纳入国家“十三五”重大水利工程规划。新连通水库泡塘14个，引蓄水3.5亿立方米，湿地补水7300万立方米，改善恢复湿地60万亩。综合治理水土流失20.3万亩。恢复芦苇湿地5万亩。大安牛心套保列入首批重点国家湿地公园。全年造林39.8万亩，三年累计造林120万亩，全面完成三年再造百万林任务，全市森林覆盖率达到12%。镇赉县被评为国家园林城。落实草原生态补奖面积780万亩，改良草场20.5万亩。新建改造人工增雨作业点30个。取缔、淘汰燃煤小锅炉296台。6个污水处理厂扩能改造、中水回用项目竣工。节能减排任务全面完成。我市被列入国家首批生态保护与建设示范区。

　　——民生保障持续增强。10个方面25项民生实事全面落实。城镇新增就业4.6万人，城镇登记失业率3.7%，解决零就业家庭比率达100%。农村劳动力转移就业39万人（次）。城乡居民最低生活保障和企业退休人员养老金稳步提高。社会医疗保险六个险种实现市级统筹。老工伤人员全部纳入工伤保险统筹管理。城乡居民社会养老保险参保率达到95%。新农合参合率达到99.4%。市区建设棚户区安置住房8782套，完成“暖房子”工程6万平方米。改造农村危房20805户。对贫困人口和贫困村进行建档立卡，实施扶贫项目197个，探索了光伏、电商等扶贫新模式，8.2万人实现脱贫。解决5.6万农村居民饮水安全。新建农村标准化学校9所，维修改造校舍1.73万平方米，通榆县、镇赉县义务教育基本均衡发展通过国家验收。申报省级科技发展计划项目87项，争取到省政府重大科技成果转化项目3项。吉林西部首家企业院士工作站落户洮北区。“歌舞鹤乡?放飞梦想”群众文化系列活动，成为国家级地方特色文化品牌。居家养老服务示范中心全面竣工。建设标准化村卫生室247所。医疗救治体系不断完善，传染病防治得到加强。地震应急指挥系统及测震虚拟台网建成。国防教育深入开展，“双十”活动扎实推进，军队保障社会化试点全面启动。人才、民族宗教、广播电视、新闻出版、体育健身、妇女儿童、外事侨务等工作取得新成绩，残疾人、老龄、人防、档案、地方志和红十字会等事业全面发展。

　　——社会大局和谐稳定。安全生产防控体系全面铺开，工矿商贸、道路交通、火灾三类事故发生起数下降29.9%，死亡人数下降55%。“信访法治建设年”活动成效明显，去省访、进京非访人次分别下降12.4%、67.4%。社会治安防控体系建设得到加强，刑事、治安案件分别下降0.5%和25.2%。“六五”普法通过省级验收。镇赉县、大安市被评为全国法治县。食品药品放心工程有力推进，保持无重大食品安全事故、无重大药品安全事件“双无”目标。公共突发事件得到有效预防和处置。

　　——自身建设不断加强。政府系统“三严三实”专题教育扎实开展，政风建设得到加强。认真办理人大代表建议和政协提案，全面落实市人大、市政协视察调研提出的意见和建议。推行政府常务会议和部门办公会议学法制度，举办科学行政讲堂9期。行政复议工作进一步加强。政务公开力度加大，行政审批3日内办结率提高到90%以上。发展软环境得到改善。审计监督机制不断完善。“三公”经费支出大幅下降。机构编制总量保持“零增长”。严格落实党风廉政建设责任制，反腐倡廉工作进一步加强。

　　各位代表！

　　这些成绩的取得来之不易，是省委、省政府高度重视、亲切关怀的结果，是市委科学决策、正确领导的结果，是市人大、市政协有效监督、大力支持的结果，是全市上下合力攻坚、拼搏奋斗的结果。在这里，我代表白城市人民政府，向全市各族人民，各民主党派、工商联、人民团体、无党派人士、离退休老同志，向驻白的中省直部门和企事业单位、人民解放军指战员、武警部队官兵、公安司法干警，向关心和支持白城发展的各界朋友，致以崇高的敬意和衷心的感谢！

　　我们既要看到成绩，更要看到前进中的困难和挑战。主要表现在：经济增长动力不足，下行压力仍然较大，企业生产经营回升缓慢；受原油产量和价格双降等不可控因素影响，财政收入增幅下降；深化改革有待于进一步推进，面对基层的矛盾和问题破解不够，体制机制不活；城市基础设施薄弱、功能不配套，与人民群众期待还有很大差距；创新社会治理更加迫切，改善民生、维护稳定任务繁重，精准扶贫攻坚需下更大力气；政府职能转变还不到位，不严不实问题还不同程度存在。我们一定直面问题，勇于担当，认真加以解决。

　　二、“十三五”时期主要目标任务

　　根据中央精神和省委要求，按照市委意见，我们编制了《白城市国民经济和社会发展第十三个五年规划纲要（草案）》，提交大会审议。

　　“十三五”时期，全市经济社会发展总体思路是：全面贯彻党的十八大和十八届三中、四中、五中全会精神，深入落实习近平总书记系列重要讲话精神，按照省委、省政府的决策部署，紧紧围绕“四个全面”战略布局，牢固树立创新、协调、绿色、开放、共享“五大发展”理念，落实“三个五”发展战略，以结构调整促转型、以改革创新添动力、以改善民生增福祉，加快建设吉林西部生态经济区，实现全面建成小康社会目标。

　　主要奋斗目标是：“两必保、三翻番”。“两必保”，就是预期性指标必保超过省和国家的高线，约束性指标必保低于省和国家的底线；“三翻番”，就是经济总量、财政收入和城乡居民收入力争翻一番。具体工作中，要紧紧围绕“一条主线”、落实“五大发展”、实现“五个突破”。

　　紧紧围绕“一条主线”：就是深入落实省委、省政府统筹东中西三大板块区域经济协调发展的要求部署，牢牢把握建设吉林西部生态经济区这条主线，保护好生态环境、开发好生态产业、建设好生态城镇、治理好生态社会。

　　落实“五大发展”、实现“五个突破”：

　　（一）落实创新发展，在激发动力活力上实现新的更大突破。深入实施创新驱动战略，让创新贯穿白城振兴发展的各领域、全过程。推动大众创业、万众创新，促进新技术、新产业、新业态蓬勃发展。进一步完善有利于激活创新潜力的体制机制和平台载体，有效发挥创新驱动的乘数效应，推动白城发展由要素驱动向创新驱动转型跨越。力争到2020年，科技综合实力达到全省中上游水平，让“白城制造”走出吉林、走向全国。

　　（二）落实协调发展，在促进统筹均衡上实现新的更大突破。坚持把协调作为推动白城持续健康发展的内在要求，更加注重城市与农村、经济与社会、人与自然协调发展。优化工业布局和城镇空间、农业空间、生态空间，统筹新型工业化、信息化、城镇化、农业现代化协同发展。着力解决区域发展不平衡、城乡二元结构等难题，在扶强弱项、补齐短板中提升整体发展水平。推动白城在“十三五”期间实现遵循经济规律的科学发展、遵循自然规律的可持续发展、遵循社会规律的包容性发展。

　　（三）落实绿色发展，在提升生态效益上实现新的更大突破。牢固树立“绿水青山就是金山银山”的理念，坚持向生态要效益、要发展，向绿色要增长点、要生产力。全面加强河湖、森林、湿地、草原等生态系统的规划和保护，大力发展绿色农业、清洁能源、生态旅游，促进生态建设与经济发展融合互促，切实提高发展质量和效益。力争到“十三五”期末，生态环境基本恢复到上世纪50年代水平，建设美丽白城。

　　（四）落实开放发展，在扩大交流合作上实现新的更大突破。抓住全省实施“双翼共进”契机，依托中蒙俄大通道建设，深度融入国家“一带一路”战略和长吉图开发开放布局。坚持走出去与引进来并重、引资和引技引智并举，不断提升对外开放的层次和水平。力争把白城打造成中蒙俄大通道的重要节点城市、面向东北亚的区域中心城市。

　　（五）落实共享发展，在增进民生福祉上实现新的更大突破。按照人人参与、人人尽力、人人享有的要求，坚持共建共享，突出普惠性、保基本、均等化、可持续，坚决打赢扶贫攻坚战，全力办好民生实事，完善社会保障体系，加强社会治理创新，不断夯实共享发展的文化基础、社会基础、法治基础、安全基础，提高公共服务的共建能力和共享水平，让全市人民享有更多获得感、幸福感，共同迈入全面小康社会。

　　三、2016年工作安排

　　2016年是“十三五”开局之年，开好局、起好步至关重要。市委五届十次全会已经描绘了新蓝图，确定了“十三五”时期指导思想和奋斗目标。这是白城未来发展的行动指南，是我们推动落实的重要遵循。政府工作总的要求是：全面贯彻落实党的十八届五中全会和省委十届六次全会、市委五届十次全会精神，坚持全面发展、争创一流，以建设吉林西部生态经济区为主线，突出生态建设、扶贫开发、经济发展、老城改造“四个重点”，全力以赴兴产业、上项目，坚定不移抓改革、促转型，持之以恒惠民生、保稳定，努力让全市人民过上更加美好的日子。

　　全市主要预期目标是：地区生产总值增长7.5%；地方级财政收入增长5.1%；固定资产投资增长13.2%；规模以上工业增加值增长8.3%；社会消费品零售总额增长10%；城镇和农村常住居民人均可支配收入分别增长7.5%和8%；城镇新增就业3.8万人，城镇登记失业率控制在4.5%以内；单位GDP能耗、二氧化碳排放强度等节能减排指标完成省下达任务。

　　重点抓好以下十个方面工作：

　　（一）强化生态白城建设。切实把生态作为立市之本，坚持以规划为引领，实施生态建设三年行动计划。一是推进河湖连通工程。着力提升建设标准，综合开发利用，三年完成泡塘连通任务，明年力争连通20个。治理水土流失面积16万亩。二是开展“造林还湿双百万”活动。三年造林100万亩、修复湿地100万亩。明年，全力申报三北百万亩防护林基地建设项目，造林35万亩。完成“三年清收、五年还林”目标。实施生态补水、退耕还湿工程，恢复湿地35万亩。推进向海、莫莫格生态移民。三是抓好草原综合治理。依法打击非法开垦草原行为，严格执行禁牧和草畜平衡制度。落实草原生态保护补奖政策，推进草原确权承包、基本草原划定。争取国家退牧还草、退耕还草试点，重点实施草原恢复治理项目，综合治理草原10万亩，形成西部优质牧草产业链。加强土地整理和复垦开发。四是着力提升环境质量。实施“蓝天工程”，推进工业企业烟尘治理，加强秸秆焚烧、扬尘、尾气等综合治理，加快供热企业整合、淘汰市区燃煤小锅炉。实施“清水工程”，推进嫩江、洮儿河等流域水污染防治，强化畜禽粪便综合治理。五是积极发展生态经济。做好生态产业发展规划，打造生态景观，发展生态旅游、林下种养、中药材、林果等产业，推动生态保护与经济发展融合互促。

　　（二）开展精准扶贫攻坚。坚持把扶贫攻坚作为最大的民生工程。按照中央“六个精准”和省委、省政府“七个一批”要求，制定扶贫攻坚三年规划，确保到2018年末实现“三确保、两不愁、三保障”目标，明年力争脱贫7.9万人。一是确定扶贫对象。组织开展建档立卡“回头看”活动，严格按照脱贫摘帽标准，逐村逐户调查核实，对已经脱贫的，从建档立卡中退出；对新识别的，单独建册、建簿、建档，确保符合条件一户不漏、不符合一户不进。二是强化扶贫措施。实施产业扶贫、生态扶贫、医疗救助、社会保障、一事一议帮扶五大扶贫工程，积极开展健康扶贫、教育扶贫行动，实行政策性兜底“托贫”。制定精准扶贫考核办法，建立正向激励机制和“脱贫摘帽”奖惩制度，探索设立扶贫专项基金，创新督查调度、考核验收和奖惩机制，促进扶贫考评与绩效考核相结合。加大对贫困少数民族乡村扶持力度。三是落实扶贫责任。健全完善扶贫开发组织机构，形成分工明确、职责明晰的责任体系。实行市、县、乡、村四级联动机制，实现机关（企事业单位）包村、干部包户两个全覆盖，做到不落一村、不落一户、不落一人。探索全民扶贫参与机制，搭建社会扶贫信息服务平台，落实扶贫公益事业税收优惠政策、各类市场主体到贫困地区投资兴业支持政策，动员驻白部队、企业院校、社会组织，以及域外白城籍成功人士参与扶贫，构建大扶贫工作格局。

　　（三）狠抓上项目增投资。坚持把投资作为稳增长、促发展的第一动力，强化项目支撑。一是加强项目储备。依托资源禀赋和优势潜力，把握国家政策导向、产业转移方向，深度谋划投资超10亿元甚至超100亿元的大项目不少于50个。建立投资项目储备库，制定三年滚动投资计划。二是突出项目招商。借鉴发达地区经验，完善招商引资政策，瞄准国际国内500强等行业领军企业，开展补链式招商，做好“无中生有”、“有中生新”两篇文章。力争全市招商引资到位资金1130亿元，增长15%。进一步理顺开发区体制机制，加快特色园区规划建设，发展“飞地经济”。各地、各开发区每年引进投资超10亿元工业项目不少于2个。明年各开发区主要指标要排在全市前列。三是强化项目建设。着力破解土地、资金等瓶颈问题，开展重点项目并联审批，落实建设条件，实施超3000万元项目300个以上，其中超亿元项目200个以上，实现竣工项目100个以上。突出高附加值的税源型项目，狠抓全产业链系列开发、梯次增值、精深加工、循环发展的工业项目，力争每年新上工业项目占全部项目的60%以上。着力推进重大基础设施项目，确保白城机场通航运营，力争嫩丹高速、白阿铁路早日竣工，加快长白铁路建设，全面开工平齐铁路电气化改造工程，启动白城至扎鲁特旗500千伏超高压汇集送出工程前期工作。

　　（四）推动工业壮大跃升。坚持把上规模、扩总量作为当前工业的主攻方向。一是发展重点产业。做强农产品加工、能源“两大支柱产业”，引进首农、泰豪等产业化龙头企业，推进400万头生猪、300万只肉羊、50万只奶山羊等养殖加工一体化项目；抓好通榆230万千瓦风电基地、镇赉众合二期生物质发电等项目，搞好全省第二批160兆瓦光伏项目建设。力争两大支柱产业总产值突破400亿元。壮大装备制造、医药健康“两大优势产业”，推进金盛太阳能硅片、天奇装备、镇赉变压器等项目建设；推动敖东药业做大做强，加快建设化学药、生物药、中成药为一体的医药及药用辅材生产基地。力争两大优势产业总产能达到200亿元。提升汽车配件、纺织服装、冶金建材、石油化工等传统产业，加快培育一批产值超百亿的大产业、纳税超亿元的大企业。二是做强现有企业。开展扶大、扶优、扶新、扶强“四扶”专项行动，支持企业扩能改造，对金塔、鑫红钻、弘泰新能源等成长性好、潜力较大的企业，着力提升技术开发、成果转化、品牌培育、商业模式创新能力，扩大企业总量，提高企业素质，增强企业核心竞争能力。三是扶持小微企业。推进大众创业、万众创新，落实发展民营经济、支持小微企业政策措施，降低市场准入门槛。建立小微企业信息库，完善服务体系建设，加强企业创业孵化载体建设，启动“助保金池”贷款业务。全年新生成小微企业350户，壮大20户小微企业升级为规模以上企业。

　　（五）发展现代农牧业。坚持用工业化思维、“互联网+”模式，放大农牧资源优势，推进农业大市、牧业强市建设。一是调整种植结构。结合玉米价格调整，适度调减普通玉米种植面积，扩大“粮改饲”试点，饲料和经济作物种植面积增加到288.5万亩，构建粮经饲三元种植结构，推进种植结构调整核心示范区建设。在城市近郊发展设施农业，探索“光伏+农业”，新建大棚（温室）2000亩。二是扩大规模经营。促进龙头企业与专业合作社、种植大户强强联合，推动土地流转150万亩，力争特色农产品基地扩大到260万亩。农业综合机械化率达到74.5%。改造提升畜禽规模养殖场，规模养殖比重达到48%。三是推动标准化生产。创建食源性食品安全生产基地，新增绿色有机无公害基地面积20万亩。推进精品畜产品生产基地建设，创建畜禽养殖标准化示范场区。建立市县乡三级农产品质量安全监管体系、检验检测体系，全力打造“有机米、健康豆、精品肉、生态鱼”品牌。四是促进全链条发展。启动农产品加工业三年提升计划，依托8个农畜产品产业园区，打造农业一二三产融合发展平台，推广“龙头企业+基地+农户”发展模式，以制度、技术和商业模式创新为动力，实现产加销一体化。五是培育新型经营主体。新发展家庭农场100个，培育典型专业合作社100个。引进和发展农业企业，新增农畜产品加工企业100户、省市级产业化龙头企业20户。培养新型职业农民2000人。

　　（六）实施服务业提升工程。突出培育生态旅游、现代物流“两大新兴产业”，促进服务业发展提速、比重提高、水平提升。一是培育生态旅游业。加强“一线八点”精品旅游线路建设，推进军事文化产业园、向海旅游公路、查干浩特环湖路等重点项目，开发打造湿地生态游、民俗风情游、军事体验游、乡村观光游品牌。力争接待游客人数、旅游收入分别增长15%和20%以上。二是提升现代物流业。提高白城内陆港规划建设标准，推动实现陆海联运，完善检验、报关、保税等功能。加快白城综合物流园、港原国际商贸城、镇赉中铁粮食物流港、洮北中北仓储物流园等项目建设，力争建设全国最大的杂粮杂豆集散地。完善商业网点布局，规范提升农贸市场。建立推动消费转型升级的长效机制。促进外贸出口取得新进展。三是发展互联网经济。建设数据中心、灾备中心和呼叫中心，抓好信息消费试点，加快白城云基地建设，推进两化融合特色产业试验区建设，大力发展“互联网+”产业。建立电子商务管理中心，建设白城特色产品O2O体验展示馆，推进国家电子商务进农村示范县项目。四是创新金融服务。推进农村金融综合改革试验区建设，构建多层次、广覆盖、可持续、风险可控的农村现代金融体系；创新符合现代农业需求的金融产品和服务；探索农村互联网金融新模式，建设普惠型金融体系；优化农业保险产品，扩大保险的范围和品种。支持中小企业开展资本市场融资。力争服务业增加值完成300.8亿元，增长7.9%。

　　（七）突出老城改造提升。围绕“一年一个变化、三年大变化、五年上层次”，开展卫生城、园林城、文明城、生态城“四城联创”活动，加强城市建设管理。一是加快改造。借助海绵城市、管廊城市试点建设机遇，集中利用两年左右时间，全力推进老城区改造攻坚。加快编制老城区改造规划。推进海绵城市建设、道路标准化和居民小区改造、“气化白城”等项目。实施环境提升工程，所有机关单位要拆墙透绿、新植增绿，向市民开放。改造建设一批城市公园、广场，努力让每个广场、小区都变成花园，让老城变新城。二是强化管理。学习借鉴先进城市管理经验，理顺市区管理体制，推行“网格化”管理，加强“智慧城市”建设。按照卫生、绿化、规划建设“三个不留死角”的要求，加强环境卫生、拆违打非、交通治理、物业管理、马路市场、乱贴乱画等专项整治，落实“门前三包”责任制。开展争做文明市民活动。三是统筹推进。抓好生态新区开发。围绕向产城融合转变、向建管并重转变，提升整体形象、提升服务功能，引进健康养老、专业物流、金融外包、电子商务等企业，推进鹤鸣湖文化创意园、吉林电子商品交易中心等项目建设。加强县城建设。争取用3年左右时间，各县（市、区）全部达到省级创建标准、至少有1个国家级称号。促进新型城镇化发展。

　　（八）深化重点领域改革。坚持把改革贯穿于发展各个环节，用改革破解瓶颈制约、激发市场活力。一是加快行政领域改革。健全简政放权、转变职能推进机制，建立权力清单、责任清单、投资负面清单、财政专项资金管理清单、政府服务网络“四个清单一张网”，推动事前审批更多地转为事中事后监管。深化行政执法体制改革。推进承担行政职能、从事生产经营活动事业单位改革。二是抓好经济领域改革。推进投融资改革，选择市政设施领域先试先行PPP模式。推动财税体制改革。改进预算控制方式，硬化预算支出约束，市本级预算单位全部纳入公务卡结算管理范围。完善国有资产管理体制。推进农村集体资产产权改革试点，农村土地确权面积达到40%。深化商事制度改革，推进“先照后证”、“五证合一”。搭建新能源交易平台，推进电力市场化交易。三是深化社会领域改革。健全科技促进农业发展机制。加快构建现代公共文化服务体系，推进文化产业发展。深化市级公立医院综合改革。完善城乡居民养老保险制度。健全价格调控机制。深入推进户籍制度改革。四是推进生态领域改革。加强生态系统的整体规划和立法保护，实施环境民事公益诉讼制度，坚守生态环境底线，划定山水林田湖生态保护红线。研究探索生态补偿机制。落实最严格的水资源管理制度。

　　（九）全力保障改善民生。坚持把新增财力的80%以上用于民生事业，努力增进人民福祉。一是办好民生实事。（1）开展城乡劳动者就业技能培训，推动就业困难人员就业4000人，农村劳动力转移就业30万人次。（2）提高居民医疗、生育保险和企业退休人员养老保险待遇标准。探索农村医保就地报销新举措。（3）结合棚户区改造，加快推进长庆北街、东风出口标准化建设。改造农村危房1万户。（4）扩能改造市区三水厂。启动农村饮水巩固提升工程。（5）加快市区至各乡镇、白城至洮南客运班线公交化改造，建设农村公路200公里。（6）新建速滑馆，推进博物馆、图书馆、群艺馆改扩建。建设城乡健身路径80个。（7）抓好老龄工作，社区居家养老中心实现全覆盖，推动公共服务场所和资源向60周岁以上老人免费开放。（8）建设农村标准化学校3所，维修改造校舍1.5万平方米。（9）城乡特困群体救助标准分别提高20%、25%。开展重大疾病医疗救助，对符合条件的救助对象实行医疗目录内费用100%救助。（10）深入实施社会主义新农村建设工程，加强村屯规划，开展农村环境连片整治，集中打造一批“美丽乡村”。二是统筹发展社会事业。加强人才队伍建设，创新人才培养、引进和使用机制。建设科技大市场。加强与大专院校、科研院所的科技合作。推进洮北、大安义务教育基本均衡发展，确保通过国家验收。拓展职教集团化办学模式。深化“歌舞鹤乡?放飞梦想”群众文化系列活动，打造文化白城。开展“快乐运动?幸福鹤乡”群众体育系列活动，举办白城市第六届全民健身运动会。搞好市中医院、120急救中心项目建设，启动社区卫生服务提升工程。全面落实两孩政策，完善计划生育服务管理。确保残疾人康复中心建成运行。做好民族宗教工作。保障妇女和未成年人权益。促进军民融合发展，抓好“双十”活动和军队保障社会化试点，争创第四次全国双拥模范城。完成第三次全国农业普查、1%人口抽样调查。继续做好三轮修志编撰。推进慈善事业发展，做好人防、侨务、档案等工作。三是维护社会安全稳定。加强安全生产“四化融合”、“三位一体”防控体系建设，坚决遏制重特大事故发生。推进依法治市，启动“七五”普法，增强全民法治意识。畅通信访渠道，完善领导接访和包案制度，切实解决好群众合理合法的利益诉求。强化平安白城建设，加大社会治安综合治理和反恐防暴、应急处突力度，严厉打击违法犯罪活动。推进食品药品综合治理，创建全国食品安全城市试点。完善应急管理综合协调机制。加强防汛抗旱、森林草原防火、地震防灾减灾等工作。

　　（十）加强政府自身建设。牢固树立宗旨意识，着力打造服务型政府。一是坚持依法行政，把政府工作全面纳入法治轨道。落实《法治政府建设实施纲要》，坚持“法无授权不可为，法定职责必须为”，严格按照法定权限和程序行使权力、履行职责。加强行政规章制度建设。完善依法决策机制，健全重大决策民意征询制度。自觉接受人大法律监督、工作监督和政协民主监督，认真办理人大代表建议和政协提案。二是坚持管理创新，着力提高行政效能。整合现有公共资源，建立政务服务软件平台，探索实行网上审批，推进行政审批事项整体提速。促进政务信息公开，有效利用微信、微博、手机客户端等新兴媒介，及时回应社会关切。推进政府向社会购买服务。加强软环境整治。三是坚持倡俭治奢，推进党风廉政建设。认真践行“三严三实”要求，严格落实中央八项规定精神，全面贯彻《廉洁自律准则》和《纪律处分条例》，坚持不懈纠正“四风”，坚定不移惩治腐败。严控“三公”经费支出。加强行政监察，发挥审计监督作用，强化对公共资金、公共资源、国有资产监管。四是坚持主动作为，切实做到勤政为民。完善绩效管理、考核评价体系，加大督查力度，健全激励机制，强化责任追究，确保政令畅通。树立“三个意识”、弘扬“三种精神”、提振“三股劲头”，勇于担责、敢于担当，一心一意谋发展，脚踏实地抓落实，保持昂扬向上、争创一流的精神状态，创造性开展工作，切实提高政府的公信力和执行力，以忠诚、干净、担当的形象取信于民。

　　各位代表！

　　全面建成小康社会的号角已经吹响，白城振兴发展的征程即将开启。让我们在省委、省政府和市委的正确领导下，在市人大、市政协的监督支持下，开拓创新，团结拼搏，为加快建设吉林西部生态经济区，让全市人民过上更加美好的日子而努力奋斗！
